# Detraining Differentially Preserved Beneficial Effects of Exercise on Hypertension: Effects on Blood Pressure, Cardiac Function, Brain Inflammatory Cytokines and Oxidative Stress

**DOI:** 10.1371/journal.pone.0052569

**Published:** 2012-12-20

**Authors:** Deepmala Agarwal, Rahul B. Dange, Jorge Vila, Arturo J. Otamendi, Joseph Francis

**Affiliations:** 1 Comparative Biomedical Sciences, School of Veterinary Medicine, Louisiana State University, Baton Rouge, Louisiana, United States of America; 2 Veterinary Clinical Sciences, School of Veterinary Medicine, Louisiana State University, Baton Rouge, Louisiana, United States of America; 3 School of Veterinary Medicine, Louisiana State University, Baton Rouge, Louisiana, United States of America; State University of Rio de Janeiro, Brazil

## Abstract

**Aims:**

This study sought to investigate the effects of physical detraining on blood pressure (BP) and cardiac morphology and function in hypertension, and on pro- and anti-inflammatory cytokines (PICs and AIC) and oxidative stress within the brain of hypertensive rats.

**Methods and Results:**

Hypertension was induced in male Sprague-Dawley rats by delivering AngiotensinII for 42 days using implanted osmotic minipumps. Rats were randomized into sedentary, trained, and detrained groups. Trained rats underwent moderate-intensity exercise (ExT) for 42 days, whereas, detrained groups underwent 28 days of exercise followed by 14 days of detraining. BP and cardiac function were evaluated by radio-telemetry and echocardiography, respectively. At the end, the paraventricular nucleus (PVN) was analyzed by Real-time RT-PCR and Western blot. ExT in AngII-infused rats caused delayed progression of hypertension, reduced cardiac hypertrophy, and improved diastolic function. These results were associated with significantly reduced PICs, increased AIC (interleukin (IL)-10), and attenuated oxidative stress in the PVN. Detraining did not abolish the exercise-induced attenuation in MAP in hypertensive rats; however, detraining failed to completely preserve exercise-mediated improvement in cardiac hypertrophy and function. Additionally, detraining did not reverse exercise-induced improvement in PICs in the PVN of hypertensive rats; however, the improvements in IL-10 were abolished.

**Conclusion:**

These results indicate that although 2 weeks of detraining is not long enough to completely abolish the beneficial effects of regular exercise, continuing cessation of exercise may lead to detrimental effects.

## Introduction

Systemic arterial hypertension is a clinical condition associated with high morbidity and mortality [1]. Hypertension is characterized by cardiac hypertrophy and dysfunction, chronic inflammation, and overactivation of the renin-angiotensin system (RAS) [2]. Though, the brain has typically been considered as a target for late stage hypertensive disease, a growing body of evidence has implicated brain in the initiation of all forms of hypertension including essential hypertension [3]. In the brain, paraventricular nucleus (PVN) is a key integrative area involved in sympathetic regulation of blood pressure (BP) and body fluid homeostasis [2], [4]–[5]. Previous reports from our laboratory and others have demonstrated that angiotensin II (AngII), a major effector peptide of the RAS, induces increased production of pro-inflammatory cytokines (PICs) [6] and oxidative stress [7]–[8] within the PVN, leading to sympathoexcitation and increased BP. PICs such as tumor necrosis factor-alpha (TNF-α) and interleukin-1β (IL-1β) act as neuromodulators and play a key role in sympathetic control of BP [6]. Recent discoveries indicate that besides elevated levels of circulating and brain PICs [6], [9]–[10], anti-inflammatory cytokines (AICs) such as IL-10 have a significant impact on sympathetic outflow, arterial pressure and cardiac remodeling in experimental models of hypertension [6]. Interestingly, it is becoming clear from all these studies that cytokines and RAS interact with each other, possibly via production of reactive oxygen species (ROS), and thereby regulate BP [6], [11]–[12]. Recent investigations have identified NADPH oxidase (NOX)-derived ROS, particularly superoxide (O2•−), as key signaling intermediates in AngII intraneuronal signaling [12]–[14]. In particular, overexpression of intracellular O2•− scavenging enzyme copper/zinc superoxide dismutase (Cu/ZnSOD) in the brain has shown to significantly inhibit the acute pressor response to centrally administered AngII [15]. Of various isoforms of NOX, the role of NOX2 (also known as gp91^phox^) in AngII-induced hypertension and endothelial dysfunction is well established [2], [16]. Besides, levels of inducible nitric oxide synthase (iNOS), another marker of oxidative stress, have been found to be dramatically upregulated in various tissues [17]–[19] including the brain [2], [20] of hypertensive animals.

Although various currently available pharmacological therapies targeting the components of the RAAS have been proven to reduce BP; the morbidity and mortality caused by hypertension is still on the rise. According to current “Heart Disease and Stroke Statistics” the death rate caused by hypertension increased 9.0% from 1997 to 2007, and the actual number of deaths increased by 35.6% [21]. Therefore, physical activity has recently been recommended as a non-pharmacological approach for the treatment and control of hypertension. Although past several years of research has proven that regular physical activity reduces BP and delays the progression of hypertension in animals and humans, the compliance with the recommended treatment has been found to be very low. For instance, non-compliance with exercise has recently been reported to be closely associated with poor outcomes of the disease [22]. When compliance to exercise was assessed in patients with controlled and uncontrolled hypertension, the authors found that 43.5% patients with controlled hypertension were compliant with exercise, whereas, only 16.7% of those with uncontrolled hypertension were compliant. Despite these alarming statistics, the effects of cessation of exercise (physical detraining) at the physiological and molecular levels in hypertension are far from understood. A few previous studies have examined the effects of detraining on heart and skeletal muscle of hypertensive and normal rats, particularly in relation to insulin sensitivity [1], [23]–[24]. However, no studies, to date, have examined the effects of detraining on inflammatory cytokines and oxidative stress, particularly, within the cardiovascular regulatory centers of the brain in hypertension. Also, the effects of detraining on cardiac morphology and function in hypertension are poorly understood.

Therefore, this study was designed to investigate the effects of detraining on mean arterial blood pressure (MAP) using radiotelemetry, and cardiac morphology and function in hypertension. We also aimed to investigate the effects of detraining on pro- and anti-inflammatory cytokines (PICs and AIC) and oxidative stress within the PVN of hypertensive rats.

## Methods

All procedures in this study were approved by the Louisiana State University Institutional Animal Care and Use Committee and were performed in accordance with the National Institutes of Health Guide for the Care and Use of Laboratory Animals.

### Animals

**Figure 1:**
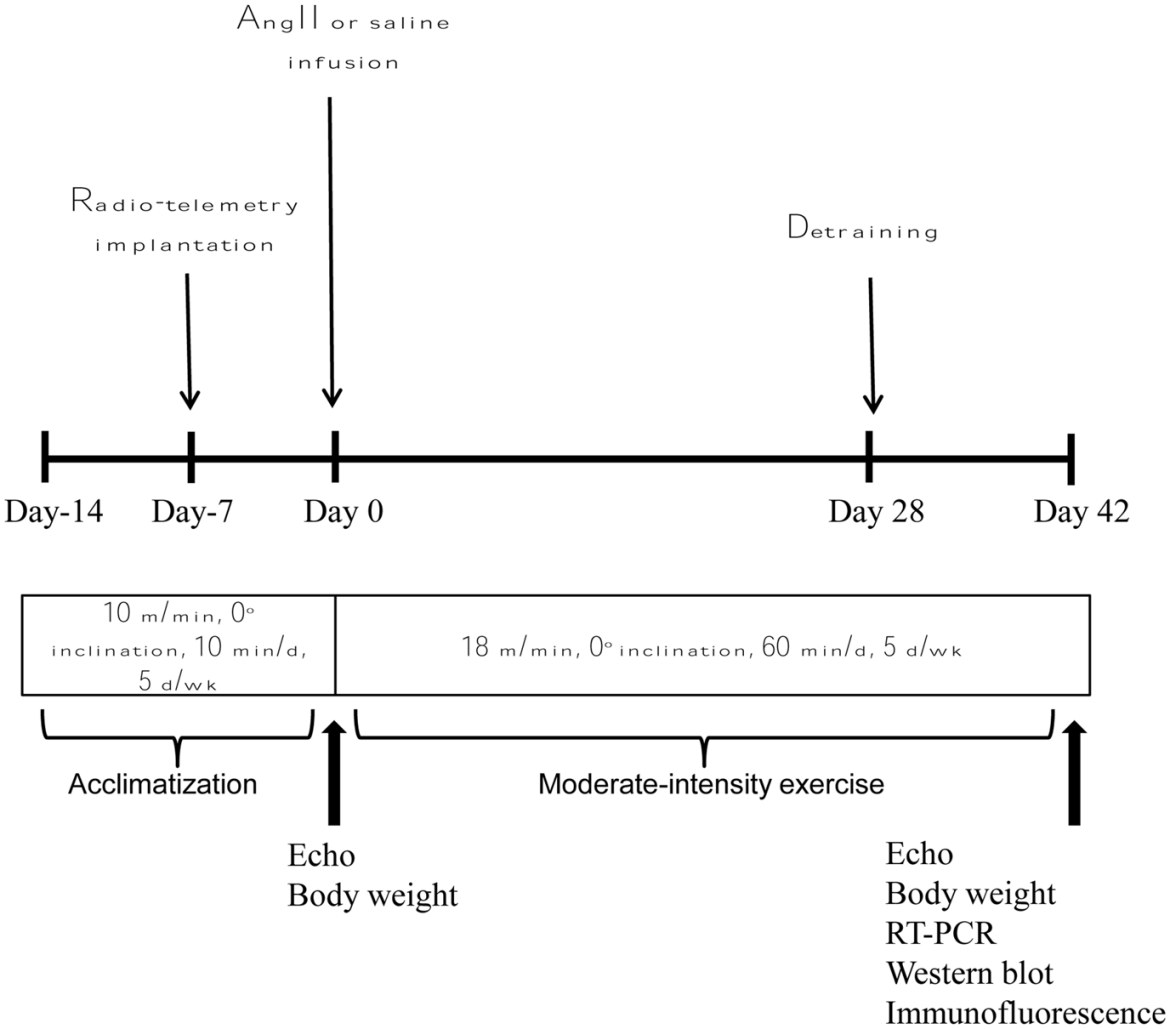
Experimental protocol. The rats were first acclimatized to the treadmill for 14 days before the start of the experiment. After 7 days of acclimation, the rats were implanted with radio-telemetry probes for continuous recording of MAP and then were allowed to recover for next 7 days. Then miniosmotic pumps (42 days) filled with AngII or saline were subcutaneously implanted. Before AngII pump implantation, animals were weighed and a baseline echocardiogram was performed. Animals in exercise groups were allowed to run for 42 days, whereas, animals in sedentary groups were placed on non-running treadmill for the exercise sessions. Animals in detraining groups underwent exercise for 28 days and were kept sedentary for the rest of the14 days. 24 hours after the last exercise session, animals were weighed and the final echocardiogram was performed. The animals were then euthanized and the brains were collected for real-time RT-PCR and Western blot analysis.

In this study, we used an Angiotensin II (AngII)-induced hypertensive rat model, a well-established model of neurogenic hypertension. A total of 90 adult male Sprague-Dawley rats (250–350 grams) were studied, of which 45 rats were infused with AngII (Bachem, CA, USA) dissolved in 0.9% saline, at a subpressor concentration of 200ng/kg/min via osmotic minipumps. This AngII dose was based on previous publications from our laboratory and others [25]. The other 45 rats were infused with saline (Sal) in place of AngII and were used as normotensive controls. The pumps were implanted subcutaneously for 42 days (6 weeks). Animals were randomized into six groups (n = 15 per group): saline+sedentary (Sal+Sed), saline+exercise (Sal+Ex), saline+detraining (Sal+Det), angiotensin II+sedentary (AngII+sed), angiotensin II+exercise (AngII+Ex), and angiotensin II+detraining (AngII+Det) (Figure 1). The animals in exercise groups were subjected to moderate intensity exercise for 42 days. Animals in detraining groups were given exercise for a period of 28 days (4 weeks) followed by 14 days (2 weeks) of detraining. Echocardiographic assessment was carried out at baseline and at the conclusion of the study. After 42 days, the rats were euthanized using CO2 inhalation; the brains were collected, and immediately frozen on dry ice. The paraventricular nucleus (PVN) tissues were punched out from the brain for further analysis.

Animals were housed in a temperature-controlled room (25±1°C) and maintained on a 12∶12 hour light:dark cycle with free access to water and food. All animal and experimental procedures were reviewed and approved by the Institutional Animal Care and Use Committee (IACUC) at Louisiana State University in compliance with NIH guidelines.

### Exercise and Detraining Protocol

Rats in exercise groups (Sal+Ex and AngII+Ex) underwent moderate-intensity exercise (5 days per week; 60 min per day at 18 m/min, 0° inclination) on a motor-driven treadmill continuously for a period of 42 days. Animals in detraining groups (Sal+Det and AngII+Det) were given moderate-intensity exercise of a period of 28 days and remained sedentary for next 14 days (i.e. detraining). All the animals were acclimatized to treadmill for 2 weeks prior to osmotic mini-pump implantation. After acclimation, training intensity was set at approximately 60% of maximal aerobic velocity (MAV), which corresponds to moderate intensity exercise (18–20 m/min). This training intensity was maintained throughout the study period. The MAV was evaluated from an incremental exercise test as reported previously [26]–[27]. The rats in sedentary groups (Sal+Sed and AngII+Sed) were placed on a nonmoving treadmill during the training sessions.

### Osmotic Minipumps Implantation

Osmotic minipumps were implanted subcutaneously under anesthesia with 2% v/v isoflurane/oxygen, 1 day before initiation of moderate-intensity exercise. The adequacy of anaesthesia was monitored by limb withdrawal response to toe pinching. After shaving of the surgical site, the skin was swabbed with povidone–iodine and alcohol, 70%. A small incision was made through the skin between the scapulae. A small pocket was formed using a hemostat to spread the subcutaneous connective tissue, and an osmotic minipump (Alzet, model 2006) with an infusion rate of 0.15 µl/h for 42 days, was inserted into the pocket, with the flow moderator pointing away from the incision. Each pump was incubated in saline, 0.9%, at 37°C for 60 h before implantation. The incision was closed with wound clips or sutures. Rats received enrofloxacin (10 mg/kg, sc) and buprenorphine (0.1 mg/kg, sc) immediately following surgery and 12 hours postoperatively.

### Blood Pressure Measurement

MAP was measured continuously in conscious rats implanted with radio-telemetry transmitters (Model TA11PA-C40, Data Sciences International, St. Paul, MN) 7 days prior to implantation of the osmotic minipumps (Figure 1). Rats (n = 6 per group) were anesthetized with a ketamine (90 mg/kg) and xylazine (10 mg/kg) mixture (ip) and placed dorsally on a heated surgical table. The adequacy of anaesthesia was monitored by limb withdrawal response to toe pinching. An incision was made on the medial surface of the left leg, the femoral artery and vein were exposed and bluntly dissected apart. The femoral artery was ligated distally, and another suture was placed proximally to temporarily interrupt the blood flow. The catheter tip of the radio-telemetry transmitter was introduced through a small hole in the femoral artery, advanced ∼6 cm into the abdominal aorta such that the tip was distal to the origin of the renal arteries, and sutured into place. The probe body was placed into the abdominal cavity and sutured to the abdominal wall. The abdominal musculature was sutured and the skin layer closed following implantation. Rats received enrofloxacin (10 mg/kg) and buprenorphine (0.1 mg/kg, s.c.) immediately following surgery and 12 hours postoperatively and allowed to recover for seven days.

### Echocardiographic Assessment of Cardiac Function and Hypertrophy

Echocardiography (n = 8 per group) was performed at baseline and at the end of the 42-day study period, as described previously [17]. Briefly, transthoracic echocardiography was performed under isoflurane anesthesia, using a Toshiba Aplio SSH770 (Toshiba Medical, Tustin, California) fitted with a PST 65A sector scanner (8 MHz probe) which generates two-dimensional images at a frame rate ranging from 300–500 frames per second. Short-axis M-mode echocardiography was performed and the following measurements were obtained as an average of at least three cardiac cycles: Left ventricular internal diameter at diastole and systole (LVIDd and LVIDs, respectively), posterior wall thickness at diastole and systole (PWTd and PWTs, respectively), interventricular septal thickness at diastole and systole (IVSd and IVSs, respectively), and fractional shortening (%FS) was calculated using the equation, FS = [(LVIDd−LVIDs)/LVIDd] X 100. Tei index was determined from left ventricular inflow and outflow Doppler recordings as previously described [28].

### Real-time RT-PCR Analysis

Semi-quantitative real-time RT-PCR (n = 9 per group) was used to determine the mRNA levels of PICs *viz.* TNF-α and IL-1β, AIC (IL-10), and oxidative stress markers *viz.* gp91^phox^ (also known as NOX2), and iNOS in the PVN by using specific primers. The primer sequences used for real-time PCR were given in Table 1. In Brief, the rats were euthanized using CO_2_ inhalation, the brains were quickly removed and immediately frozen on dry ice. The brains were blocked in the coronal plane, sectioned at 100 µm thickness, and the PVN were punched from each brain according to the methods described by Palkovits and Brownstein [29]. Total RNA isolation, cDNA synthesis and RT-PCR were performed as previously described [2]. Gene expression was measured by the ΔΔCT method and was normalized to GAPDH mRNA levels. The data is presented as the fold change of the gene of interest relative to that of control animals.

**Table 1:** Rat primers used for real-time RT-PCR.

Gene	Sense	Antisense
GAPDH	agacagccgcatcttcttgt	cttgccgtgggtagagtcat
TNF-α	gtcgtagcaaaccaccaagc	tgtgggtgaggagcacatag
IL-1β	gcaatggtcgggacatagtt	agacctgacttggcagaga
IL-10	gggaagcaactgaaacttcg	atcatggaaggagcaacctg
gp91^phox^	cggaatctcctctccttcct	gcattcacacaccactccac
iNOS	ccttgttcagctacgccttc	ggtatgcccgagttctttca

IL, Interleukin; TNF-α, Tumor necrosis factor-alpha; gp91^phox^, NADPH oxidase subunit; iNOS, Inducible nitric oxide synthase; GAPDH, Glyceraldehyde 3-phosphate dehydrogenase.

### Western Blot Analysis

The tissue homogenates from the PVN were subjected to Western blot analysis (n = 6 per group) for the determination of protein levels of PICs (TNF-α, IL-1β), IL-10, gp91^phox^, iNOS, Cu/ZnSOD, and GAPDH. The extraction of protein and Western blot was performed as described before [2]. Specific antibodies used included: TNF-α, IL-1β, gp91^phox^, iNOS, Cu/ZnSOD and GAPDH, at 1∶1,000 dilution; and IL-10, at 1∶500 dilution. Antibodies were commercially obtained: TNF-α (Abcam Inc, MA, USA); IL-1β, iNOS, and GAPDH (Santa Cruz Biotechnology, Santa Cruz, CA, USA); IL-10 (Abbiotec, CA,USA); gp91^phox^ (BD biosciences, USA); and Cu/ZnSOD (EMD Millipore, MA, USA). Immunoreactive bands were visualized using enhanced chemiluminescence (ECL Plus, Amersham), band intensities were quantified using Versa Doc MP 5000 imaging system (Bio-Rad), and were normalized with GAPDH.

### Statistical Analysis

All data are presented as mean±SE. For all the parameters except blood pressure data, statistical analysis was done by one-way ANOVA with a Tukey’s post hoc test for multiple comparisons. Blood pressure datawere analyzed by two-way repeated-measures ANOVA with a Tukey’s post hoc test. *P*-value less than 0.05 was considered statistically significant. Statistical analyses were performed using Prism (GraphPad Software, Inc; version 5.0).

## Results

### Baseline Characteristics

Table 2 shows the baseline characteristics of the studied animals. At the beginning of the study, the body weight and echocardiographic parameters were similar between groups and all rats had normal MAP.

**Table 2:** Baseline characteristic of studied rats: BW, MAP, and Echocardiographic Analysis of Cardiac Hypertrophy and Function.

Parameters	Sal+Sed	Sal+Ex	Sal+Det	AngII+Sed	AngII+ExT	AngII+Det
BW (g)	270.7±7.5	269.8±4.8	270.0±5.0	272.9±1.7	271.0±6.4	273.0±6.2
MAP (mmHg)	109.0±1.9	105.6±2.2	106.5±2.0	103.6±5.4	102.3±2.8	105.3±2.8
IVSTd, mm	1.7±0.04	1.6±0.03	1.6±0.02	1.7±0.03	1.6±0.04	1.6±0.05
IVSTs, mm	2.9±.12	2.6±0.06	2.6±0.02	2.9±0.05	2.7±0.07	2.9±0.06
LVIDd, mm	7.4±0.13	7.5±0.17	7.7±0.15	7.5±0.16	7.5±0.07	7.3±0.18
LVIDs, mm	4.2±0.14	4.2±0.10	4.3±0.09	4.4±0.15	4.3±0.11	4.0±0.09
LVPWTd, mm	1.6±0.06	1.6±0.05	1.6±0.04	1.7±0.06	1.5±0.06	1.6±0.11
LVPWTs, mm	2.6±0.06	2.8±0.13	2.9±0.16	2.8±0.05	2.7±0.15	2.7±0.14
FS, %	43.4±2.3	44.1±0.5	44.0±0.7	42.7±1.0	42.8±1.7	44.8±0.8
EF, %	77.0±1.2	80.2±1.8	80.4±1.0	77.5±2.6	80.0±1.8	82.8±2.2
HR (bpm)	356±3	358±5	354±5	344±7	361±6	365±6
Tei index	0.516±0.06	0.494±0.04	0.486±0.01	0.564±0.03	0.514±0.04	0.414±0.02

Values are mean ±SE. Sal+Sed, saline+sedentary; Sal+Ex, saline+exercise; Sal+Det, saline+detraining; AngII+Sed, angiotensionII+sedentary; AngII+Ex, angiotensinII+exercise; AngII+Det, angiotensinII+detraining. BW(g), body weight (grams); MAP, mean arterial pressure (mmHg). LVIDd and LVIDs indicate left ventricular internal diameter at diastole and systole, respectively; IVSTd and IVSTs, interventricular septal thickness at diastole and systole, respectively; LVPWTd and LVPWTd, left ventricle posterior wall thickness at diastole and systole, respectively; FS, fractional shortening (%); EF (%), ejection fraction; HR, heart rate; bpm, beats per minute.

### Effects of Exercise and Detraining on MAP

**Figure 2:**
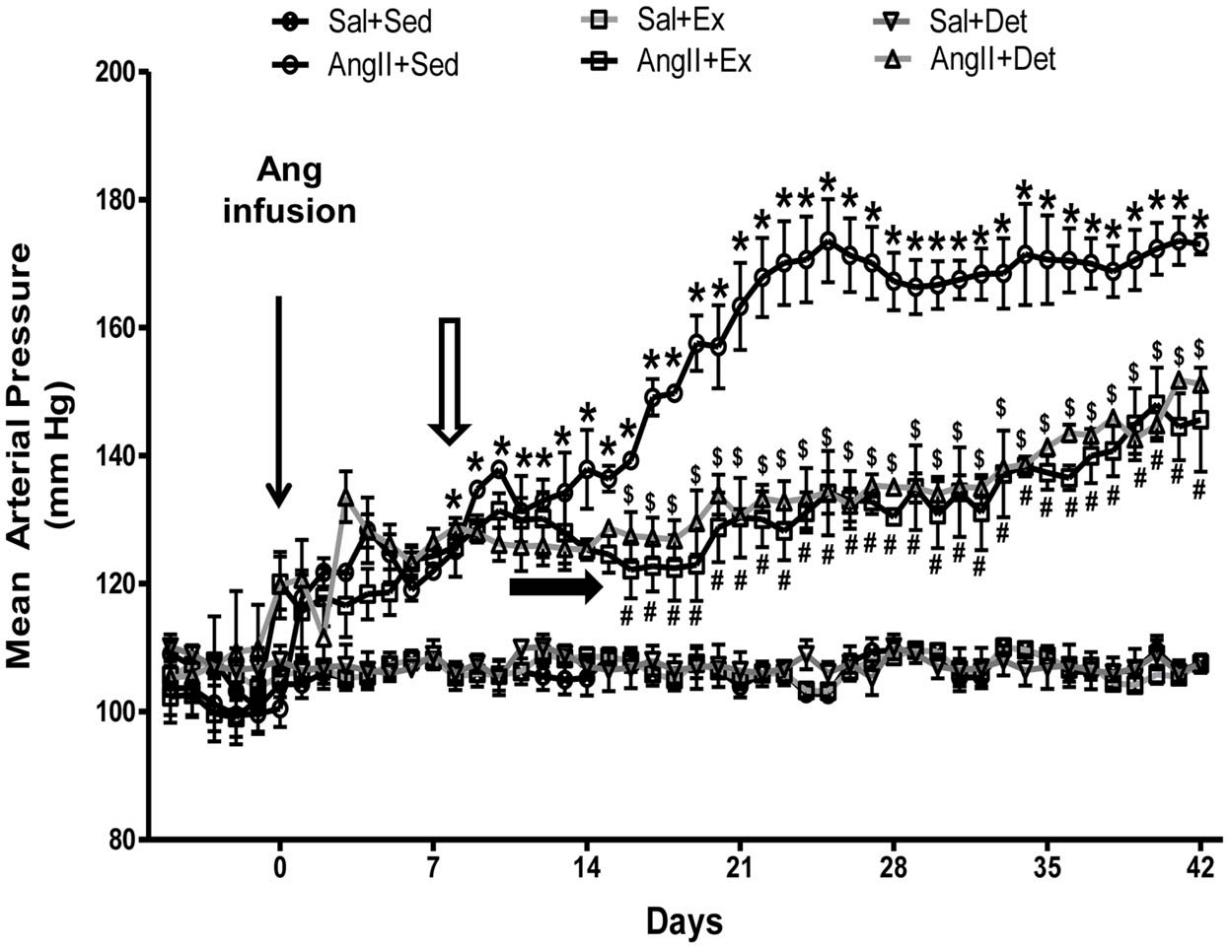
Time course of mean arterial pressure (MAP, in millimeters of mercury) in normotensive and hypertensive rats. MAP was significantly increased in AngII+Sed compared with Sal+Sed rats from day 8 of AngII infusion (empty arrow). MAP was significantly reduced in AngII+Ex compared with AngII+Sed rats from day 16 of exercise (filled arrow). 2 weeks of detraining did not abolish the exercise-induced reduction in MAP in AngII-infused rats. Values are mean±SE; n = 6 per group. *p*<*0.05 Sal+Sed versus AngII+Sed; ^#^p<0.05 AngII+Sed versus AngII+Ex; ^$^p<0.05 AngII+Sed versus AngII+Det.

As shown in Figure 2, AngII infusion in sedentary rats caused significant increase in MAP starting at day 8 of infusion when compared to Sal+Sed and remained significant for the duration of the study. The maximum increase in MAP in AngII+Sed rats was observed at day 23 of infusion after which it reached a plateau. Regular exercise prevented AngII-induced increase in MAP and in comparison with AngII+Sed, the MAP was found to be significantly lower in AngII+Ex rats beginning from day 16 of exercise when compared to AngII+Sed rats. Similarly, in AngII+Det group, exercise caused significant reduction in MAP beginning from day 16 when compared with AngII+Sed. There was no difference in MAP between AngII+Ex and AngII+Det rats. Exercise did not affect MAP in normotensive rats.

### Effects of Exercise and Detraining on Cardiac Hypertrophy and Cardiac Function

**Figure 3:**
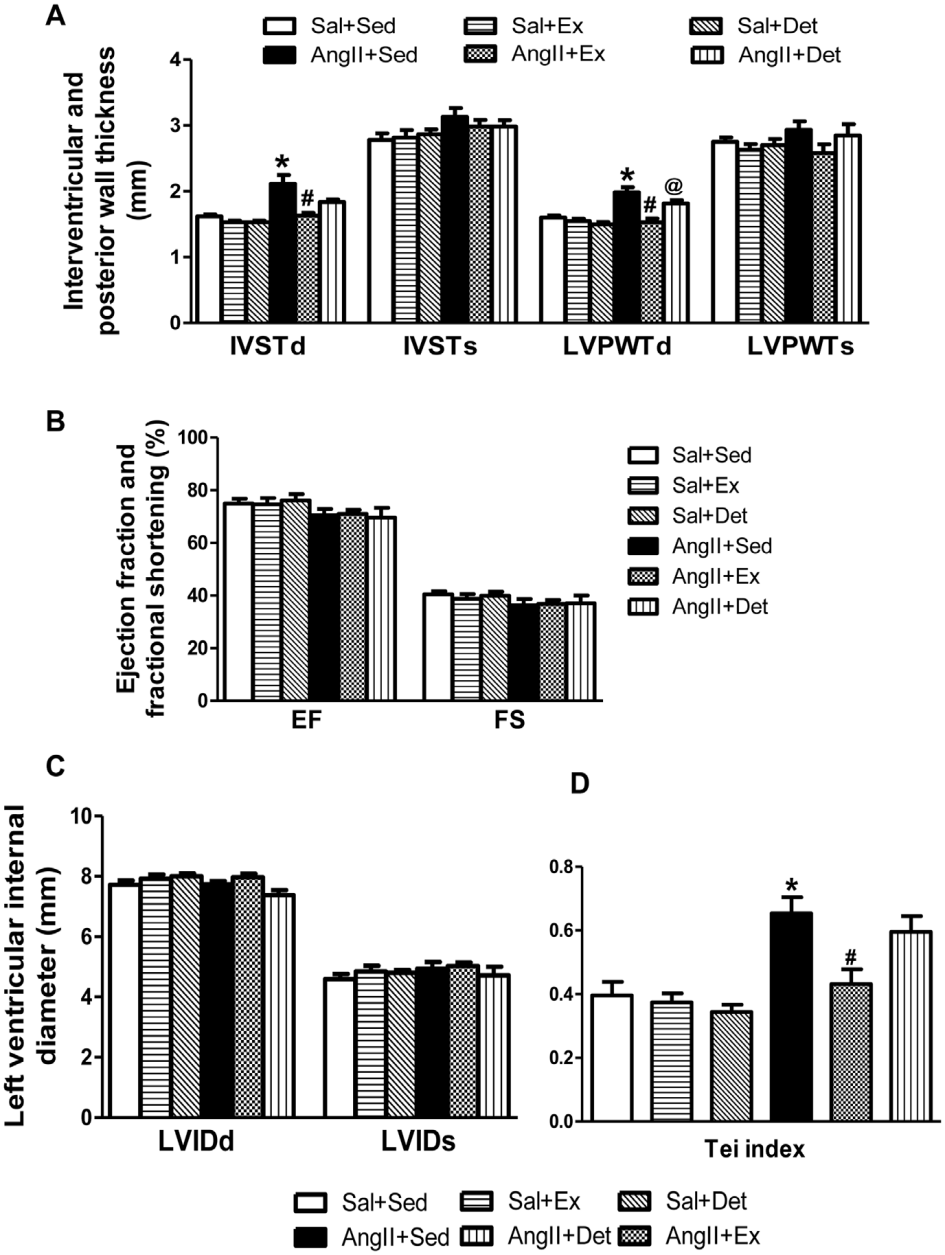
Effect of exercise and detraining on cardiac hypertrophy and cardiac function in normotensive and hypertensive rats as measured by M-mode and Doppler echocardiography. AngII+Sed rats had significantly higher levels of IVSTd, LVPWTd, and Tei index when compared to Sal+Sed. Exercise caused significant reduction in these variables in AngII+Sed rats. 2 weeks of detraining resulted in significantly increased LVPWTd in comparison with AngII+Ex; whereas, IVSTd and Tei index values were considerably but insignificantly increased in AngII+Det versus AngII+Ex. These data suggest that detraining caused partial reversal of exercise-induced changes in hypertensive rats. Values are mean±SE. n = 8 per group. *p*<*0.05 Sal+Sed versus AngII+Sed; ^#^p<0.05 AngII+Sed versus AngII+Ex; ^@^p<0.05 AngII+Ex versus AngII+Det.

At the end of the study period, AngII+Sed had higher heart mass (HM) and HM:BW ratio compared with Sal+Sed rats (Table 3). Echocardiographic studies (Figure 3A–C) revealed that when compared with Sal+Sed, AngII+Sed rats had significantly higher interventricular septal thickness (IVSTd) and left ventricular posterior wall thickness at diastole (LVPWTd), without modification of LV chamber size. These echocardiographic changes indicate the presence of concentric cardiac hypertrophy and suggest diastolic dysfunction in AngII-induced hypertensive rats. Furthermore, the increased Tei index (Figure 3D) in AngII+Sed when compared with Sal+Sed rats confirms the presence of diastolic dysfunction in hypertensive rats. AngII+Ex rats had significantly reduced HM:BW ratio, IVSTd, LVPWTd, and Tei index when compared to sedentary hypertensive rats, indicating attenuated cardiac hypertrophy and improved diastolic function in trained animals.

**Table 3:** Effect of Exercise and Detraining on Weights, MAP, and HR of rats.

Parameters	Sal+Sed		Sal+Ex	Sal+Det	AngII+Sed		AngII+Ex		AngII+Det	
BW (g)	368.9±9.1	386.6±5.9		380.8±6.0	383.8±6.4	373.8±8.9		373.7±10.7		
HM (g)	1.124±0.04	1.219±0.03		1.180±0.04	1.473±0.07*	1.354±0.10		1.214±0.04		
HM/BW (mg/g)	3.09±0.04	3.02±0.08		3.10±0.02	3.70±0.12*	3.11±0.08#		3.30±0.20		
MAP (mmHg)	107.5±0.80	108.0±0.6		108.5±0.7	173.0±1.6*	145.6±8.1#		151.2±0.61		
HR (bpm)	357±10	331±4		351±4	355±10	330±5		347±9		

Values are mean ±SE. Sal+Sed, saline+sedentary; Sal+Ex, saline+exercise; Sal+Det, saline+detraining; AngII+Sed, angiotensinII+sedentary; AngII+Ex, angiotensinII+exercise; AngII+Det, angiotensinII+detraining. BW(g), body weight (grams); HM (g), heart mass (grams); HM/BW (mg/g), heart mass to body weight ratio; MAP, mean arterial pressure (mmHg); HR, heart rate; bpm, beats per minute.

* p<0.05 Sal+Sed vs AngII+Sed;

# p<0.05 AngII+Sed vs AngII+Ex.

Interestingly, there was no significant difference in IVSTd and Tei index between AngII+Det and AngII+Ex; however, the values were slightly higher in AngII+Det when compared to AnII+Ex rats. Additionally, there was no significant difference in IVSTd and Tei index between AngII+Det and AngII+Sed. However, The LVPWTd was significantly increased in the AngII+Det rats when compared to AngII+Ex, and there was no difference when compared to the AngII+Sed rats. AngII+Det rats had significant increase in LVPWTd and a slight but insignificant increase in IVSTd and Tei index when compared to AngII+Ex, suggesting that 2 weeks of detraining may not be sufficient to completely reverse the exercise-induced changes in cardiac hypertrophy and function but it may lead to complete reversal if continued for longer than 2 weeks.

### Effects of Exercise and Detraining on Pro-inflammatory Cytokines in the PVN of Hypertensive Rats

**Figure 4:**
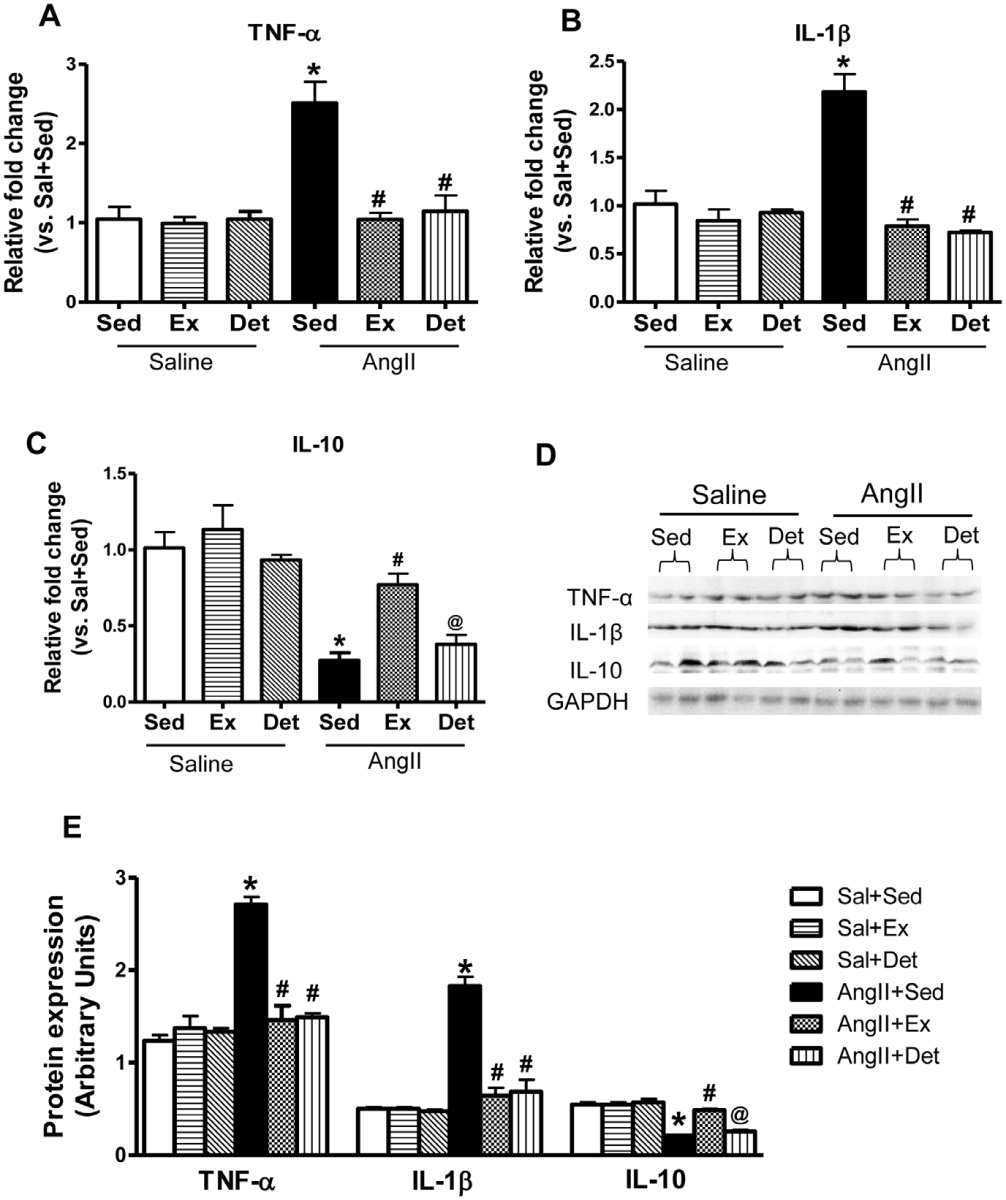
Effects of exercise on TNF-α, IL-1β, and IL-10 in the PVN of normotensive and hypertensive rats. **A,** mRNA expression of TNF-α. **B,** mRNA expression of IL-1β. **C,** mRNA expression of IL-10. **D**, a representative Western blot. **E,** densitometric analysis of protein expression. Detraining did not alter exercise-induced reduction in TNF-α and IL-1β levels in the PVN of AngII-infused animals; whereas, it did abolish exercise-mediated increase in IL-10 levels.Values are mean±SE. n = 9 per group for mRNA and n = 6 per group for protein analysis. *p*<*0.05 Sal+Sed versus AngII+Sed; ^#^p<0.05 AngII+Sed versus AngII+Ex and AngII+Sed versus AngII+Det; ^@^p<0.05 AngII+Ex versus AngII+Det.

To investigate the influence of exercise and detraining on PICs within the PVN of hypertensive rats, we examined the mRNA (Figure 4A–B) and protein (Figure 4D–E) levels of TNF-α and IL-1β. AngII+Sed rats exhibited significant increases in TNF-α and IL-1β expression in the PVN compared to Sal+Sed. This upregulation was significantly attenuated by regular exercise in AngII-induced hypertensive rats. Interestingly, two weeks of detraining did not reverse the effects of exercise on PICs. There was a significant difference in TNF-α and IL-1β levels between AngII+Sed and AngII+Det rats, while, there was no difference in AngII+Ex and AngII+Det groups.

### Effects of Exercise and Detraining on Anti-inflammatory Cytokines in the PVN

To investigate the influence of exercise and detraining on anti-inflammatory status within the PVN, we determined the mRNA (Figure 4C) and protein (Figure 4D–E) levels of IL-10, a potent AIC. IL-10 levels were significantly lowered in the PVN of AngII+Sed when compared with Sal+Sed rats. Regular exercise resulted in significant upregulation of IL-10 levels in AngII-induced hypertensive rats as indicated by significantly increased IL-10 levels in AngII+Ex rats when compared with AngII+Sed. Interestingly, IL-10 levels in AngII+Det group were significantly lower than the AngII+Ex and they were not significantly different from the AngII+Sed group.

### Effects of Exercise and Detraining on Oxidative Stress in the PVN

**Figure 5:**
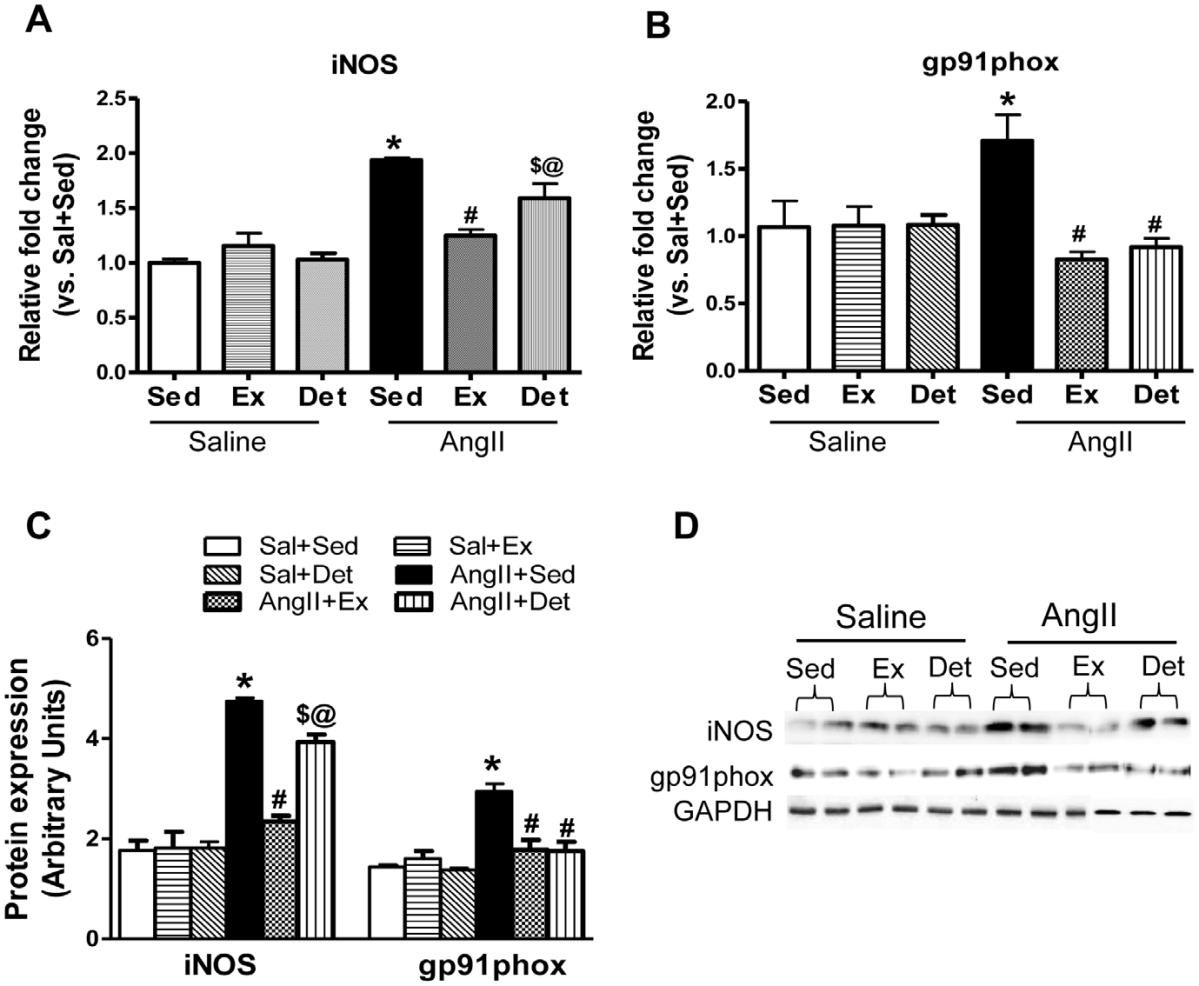
Effects of exercise on iNOS and gp91^phox^ in the PVN of normotensive and hypertensive rats. **A,** mRNA expression of iNOS. **B,** mRNA expression of gp91^phox^. **C,** a representative Western blot. **D**, densitometric analysis of protein expression. Detraining did not alter exercise-induced reduction in gp91^phox^ levels in the PVN of AngII-infused animals; whereas, it partially abolished exercise-mediated reduction in iNOS levels.Values are mean±SE. n = 9 per group for mRNA and n = 6 per group for protein analysis. *p*<*0.05 Sal+Sed versus AngII+Sed; ^#^p<0.05 AngII+Sed versus AngII+Ex and AngII+Sed versus AngII+Det; ^@^p<0.05 AngII+Ex versus AngII+Det; ^$^AngII+Sed versus AngII+Det.

To assess whether training and detraining can modulate oxidative stress within the PVN, we examined the expression levels of gp91^phox^, (a subunit of NADPH Oxidase, major source of AngII-induced ROS production) and inducible NOS (iNOS). Both protein and gene expression levels of iNOS (Figure 5A, C–D) were significantly elevated in AngII+Sed when compared to Sal+Sed rats. Exercise caused significant reduction in iNOS expression in the PVN of hypertensive rats. Importantly, iNOS levels in AngII+Det group were significantly higher than the AngII+Ex rats.

Similarly, as shown in Figure 5B–D, gp91^phox^ expression was significantly higher in AngII+Sed than Sal+Sed rats within the PVN. Whereas, when compared to AngII+Sed, AngII+Ex rats had significantly reduced levels of gp91^phox^ expression in the PVN. A similar reduction was observed in AngII+Det compared to AngII+Sed group. Among hypertensive rats, there were no significant differences in gp91^phox^ expression between detraining and exercise group.

**Figure 6:**
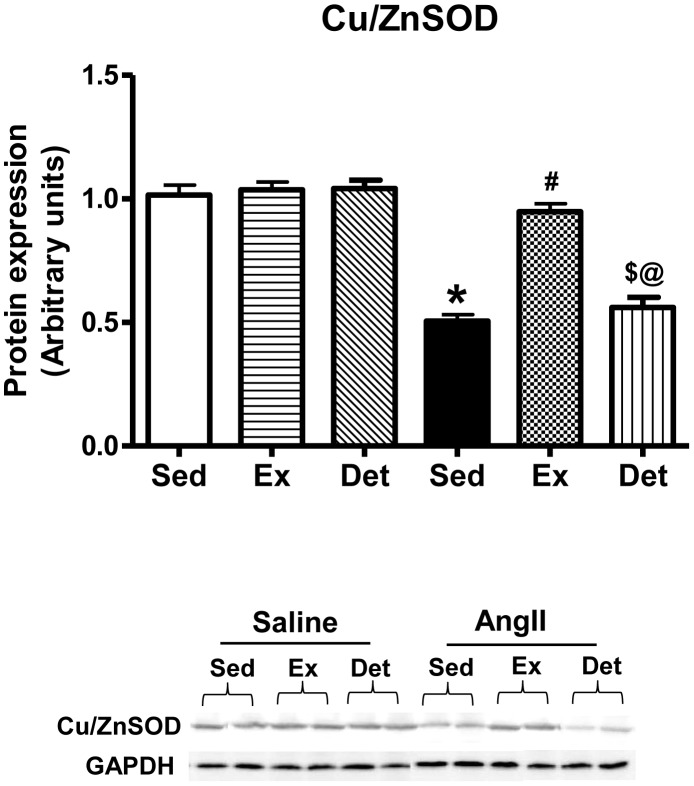
Effects of exercise on Cu/ZnSOD in the PVN of normotensive and hypertensive rats. Densitometric analysis of protein expression (upper panel) and a representative Western blot (lower panel) showed that detraining completely abolished exercise-mediated increase in Cu/ZnSOD levels.Values are mean±SE. n = 6 per group. *p*<*0.05 Sal+Sed versus AngII+Sed; ^#^p<0.05 AngII+Sed versus AngII+Ex and AngII+Sed versus AngII+Det; ^@^p<0.05 AngII+Ex versus AngII+Det; ^$^AngII+Sed versus AngII+Det.

Because decreased local antioxidant protection is one of the potential sources of ROS formation [30], we analyzed protein expression of Cu/ZnSOD, a potent superoxide scavenging enzyme (Figure 6). We observed that AngII+Sed rats had significantly increased levels of Cu/ZnSOD when compared with Sal+Sed rats. Whereas, AngII+Ex rats had significantly increased Cu/ZnSOD expression in comparison with AnII+Sed, indicative of improvements in antioxidant defense by exercise training. Interestingly, AngII+Det rats exhibited significant reduction in Cu/ZnSOD levels when compared to AngII+Ex; whereas, there was no significant difference between AngII+Sed and AngII+Det. These findings suggest that 2 weeks of detraining causes reversal of exercise-induced improvement in antioxidant status within the PVN. Exercise or detraining did not affect Cu/ZnSOD levels in normotensive rats.

## Discussion

The present study sought to evaluate the impact of regular exercise and 2 weeks of detraining on blood pressure, cardiac hypertrophy and cardiac function in an AngII-induced hypertensive rat model. Also, we investigated the impact of exercise and detraining on pro- and anti-inflammatory cytokines and oxidative stress within the PVN of these hypertensive rats. Three novel and important findings emerge from this study. First, two weeks of detraining did not abolish the exercise-induced attenuation in MAP in hypertensive rats, whereas, detraining failed to completely preserve the exercise-mediated improvement in cardiac hypertrophy and diastolic function in these rats. Second, two weeks of detraining did not have any detrimental effects on exercise-induced improvement in PICs; whereas, it abolished the exercise-induced improvement in IL-10 in the PVN of hypertensive rats. Third, 2 weeks of detraining in exercising hypertensive rats abolished the exercise-induced attenuation in oxidative stress within the PVN, as indicated by increased levels of iNOS as well as reduction in Cu/ZnSOD after detraining. Collectively, these results led us to conclude that 2 weeks of detraining is not long enough to completely abolish the exercise-induced beneficial effects; however, further cessation of exercise may lead to complete reversal of the beneficial effects.

It is now well established that an overactivation of the RAS within the brain plays a key role in the pathogenesis of hypertension. AngII, which is a major effector molecule of the RAS, induces vasoconstriction, aldosterone secretion, increased sympathetic activity and sodium retention, ultimately leading to increased BP [31]. Over time, sustained elevation of AngII leads to cardiac hypertrophy and remodeling, further deteriorating the hypertensive condition [31]. Previous findings from our laboratory and others have shown that blockade of vasoconstrictor components of the RAS [32] or overexpression of vasoprotective components of the RAS [8], [33] within the cardiovascular regulatory centers of the brain (such as PVN) attenuates BP, reduces cardiac hypertrophy and improves cardiac function in animal models of hypertension. These reports emphasize the importance of blocking RAS specifically within the PVN in mitigating the hypertensive response and associated cardiac damage. Therefore, the results of the present study are important from clinical perspective as they demonstrate that regular exercise not only reduces BP and improves cardiac function but also reduces inflammatory cytokines and oxidative stress within the PVN of hypertensive animals. Additionally, our current finding that the transient cessation of exercise could reverse exercise-induced beneficial effects in hypertension further emphasizes the importance of regular exercise in attenuating hypertension.

At the end of the study, we observed significant reduction in MAP in trained hypertensive rats compared with their sedentary counterparts and saw no comparable changes in trained normotensive controls. As depicted in Figure 2, the continuous recording of MAP in conscious rats by implanted telemetry device showed that AngII infusion resulted in significant increase in MAP in sedentary rats beginning from day 8 of infusion and this increase in MAP reached to plateau at day 23 of infusion. Regular exercise resulted in significant reduction in MAP beginning from day 16 of training and remained significantly lower until the end of the study. These results are in accordance with previous findings from our laboratory and others [17], [27]. It is noteworthy that the exercise training protocols presently used did not completely normalize the increased MAP in hypertensive rats. However, intensity, frequency, duration, and type of exercise have previously shown to affect the magnitude of the BP reduction in hypertensive animals and humans [27], [34]–[36]. Therefore, future studies are still warranted to determine which is the best exercise training intensity or frequency to completely normalize BP. Nevertheless, the results of the present study suggest that regular exercise delays the progression of hypertension. This finding is significant from a clinical perspective, because evidence suggest that a reduction of BP by only 5 mmHg can significantly reduces the risk of stroke, heart failure, and mortality from cardiovascular diseases [37]. Interestingly, 2 weeks of detraining preceded by 4 weeks of exercise in AngII-induced hypertensive rats was found to be insufficient to abolish exercise-induced attenuation in MAP as indicated by no significant difference in MAP between AngII+Ex and AngII+Det rats. In accordance with these findings, previous reports have demonstrated that 10 weeks of exercise attenuated BP in spontaneously hypertensive rats (SHRs) and 1 or 2 weeks of detraining did not affect attenuated BP in these rats [1]. It is noteworthy that previous studies from our lab and others have used tail-cuff method for BP measurements and most of those studies reported BP as measured only before and/or after the study. Whereas, to best of our knowledge, this is the first study that has employed telemetry recording of MAP in conscious sedentary and exercising animals without causing any undue stress on animals. This methodological improvement in the present study not only allowed us to obtain the most accurate measurements but also allowed us to monitor day-to-day changes in BP in relation to exercise and detraining. Nonetheless, the data suggests that although two weeks of detraining may not be long enough to revert MAP back to sedentary values, continuing detraining may lead to complete reversal.

Our echocardiographic data showed that regular moderate-intensity exercise resulted in reduced cardiac hypertrophy and improved diastolic function in hypertensive rats Interestingly, 2 weeks of detraining failed to completely preserve this exercise-induced improvements in cardiac hypertrophy and function as suggested by significant increase in LVPWTd and a not significant but considerable increase in IVSTd and Tei index in AngII+Det when compared to AngII+Ex rats. These results extended the observations of Bocalini *et al*, who demonstrated that 2 weeks of detraining was sufficient to reverse LVPWT in healthy female rats [38]. However, our study examined in detail cardiac function using M-mode and Doppler echocardiography performed in the same animal at baseline and at the end of the study, thus providing greater insight into the effects of detraining on cardiac function and morphology.

In the present study, the detraining could not fully preserve the cardioprotective effects of exercise; however, it is noteworthy that the 2 weeks of detraining was not sufficient to completely reverse the benefits either. Therefore, it is plausible to suggest that cessation of exercise for more than 2 weeks may lead to complete reversal of the cardioprotection offered by regular exercise. In support of this, it has previously been reported that resting cardiac output is reduced in trained SHRs, and that it returns to sedentary values only after 5 weeks of detraining [39]. Additionally, 5 weeks of detraining in these SHRs led to reversal of resting HR and peripheral vascular resistance to pre-training levels [39]. Furthermore, Mostarda *et al*. [40] has also demonstrated that 3 weeks of detraining did not cause reversal of hemodynamic benefits in diabetic animals. Taken together, the current findings along with previous studies clearly suggest that shorter periods of detraining may prove to be insufficient in abolishing the beneficial effects of exercise in hypertension. Continued absence of exercise can certainly have detrimental effects and hence emphasis should be given to regular active life-style to maintain the benefits.

Besides cardiac hypertrophy and diastolic dysfunction, hypertension is characterized by chronic inflammation which is reflected by a two- to threefold increase in circulating levels of several PICs [9]. In addition, the past few years of research have implicated brain cytokines, particularly in the PVN of the brain, in the pathogenesis of hypertension as well. It is apparent from these studies that PICs such as TNF-α and IL-1β act as neuromodulators and play a pivotal role in sympathetic regulation of BP [6]. For instance, an increased levels of PICs such as TNF-α and IL-1β have been found in the PVN of hypertensive rats [2], [7]. Moreover, infusion of IL-1β intracerebroventricularly [41]–[42] or microinjection into the PVN [43] increases sympathetic activity and resting arterial BP in conscious animals. Additionally, anti-inflammatory cytokines (AIC) such as IL-10 have a significant impact on arterial pressure [6]. IL-10 is known to exert inhibitory effects on PICs in the peripheral immune system and it also has a similar role in the CNS [44]. Overexpression of IL-10 in the brain (particularly within the PVN) ameliorates hypertension and associated organ damage in hypertensive rats [45]–[46]. We have recently reported that chronic regular exercise of 16 weeks duration decreases PICs and upregulates IL-10 levels in the brain of SHRs [2]. In the present study, we found that regular exercise induces similar improvements in PIC and AIC in the PVN of AngII-induced hypertensive rats. Interestingly, 2 weeks of detraining did not abolish the exercise-mediated improvement in TNF-α and IL-1β levels in the PVN. In contrast, detraining reversed the IL-10 levels back to near sedentary values in hypertensive rats. Given that it is not only the PICs but the balance between PIC and AIC that determines the outcome of the disease, there is a possibility that the reduction of IL-10 levels by detraining may ultimately lead to upregulation of PICs, if continued longer than 2 weeks. Nevertheless, our data suggest that the anti-inflammatory defense system of the body is vulnerable and sensitive to detraining. These data also emphasize the importance of regular physical activity in improving the anti-inflammatory status in hypertension.

Research over past several decades has established that PICs contribute to the AngII-induced increase in BP via induction of oxidative stress [12], [47]. AngII is a potent activator of NADPH oxidase (NOX), a primary source of reactive oxygen species (ROS), particularly the superoxide anion (O_2_^−^) [48]. NOX-derived ROS acts as potent intra- and intercellular second messengers in signaling pathways causing hypertension [13]. Of the various isoforms of NOX, the role of NOX2 (gp91^phox^) in AngII-induced hypertension is well established [16]. Activity and expression of gp91^phox^ within the cardiovascular regulatory centers of the brain has been shown to be increased in various rat models of hypertension [2], [49]. Recent reports also showed that the AngII-induced increase in BP and cardiac damage is attenuated by treatment with NOX inhibitors or Tempol, an O_2_^−^scavanger [20], [49]. Given the role of AngII-induced oxidative stress within the brain in hypertension, it is interesting to investigate whether training and detraining has the ability to influence ROS generation within the brain of hypertensive rats. Our data illustrates that regular exercise dramatically downregulated gp91^phox^ and iNOS levels and significantly improved Cu/ZnSOD levels in hypertensive rats, suggesting attenuated oxidative stress. Although not in the brain, similar increases in SOD expression have previously been shown in heart and thoracic aorta of exercising rats [50]. Interestingly, 2 weeks of detraining abolished the effects of exercise on iNOS; whereas, gp91^phox^ levels remained unchanged in detrained animals when compared with trained hypertensive rats. These changes were associated with complete reversal of exercise-induced improvement in Cu/ZnSOD in detrained animals. Taken together, these results indicate that 2 weeks of detraining abolishes the exercise-induced reduction in oxidative stress within the PVN of hypertensive rats.

Previous studies have investigated the effects of detraining on heart and skeletal muscle of hypertensive and normal rats in relation to insulin sensitivity [1], [23]–[24]. For instance, 48 hours [23] to 1 week [24] of detraining was found to reduce GLUT4 gene expression in the skeletal muscle of normotensive rats. In another study, cessation of training for 1 week resulted in reduced levels of GLUT4 in the heart and white fat tissue in both normotensive and hypertensive rats [1]. However, to the best of our knowledge, the present study is the first to demonstrate the effects of detraining on inflammatory cytokines and oxidative stress, in particular within the brain of AngII-induced hypertensive animals. Also, the effects of detraining on cardiac morphology and function in hypertension have rarely been studied before.

In summary, this study demonstrated that 2 weeks of detraining could partially revert the exercise-induced improvements in cardiac hypertrophy, cardiac function, anti-inflammatory cytokine (IL-10) and oxidative stress in the PVN of hypertensive rats, although, positive effects in MAP and PICs remained unchanged. These results indicate that although 2 weeks of detraining is not long enough to completely abolish the beneficial effects of regular exercise, continuing cessation of exercise may lead to detrimental effects.

### Perspectives

**Figure 7:**
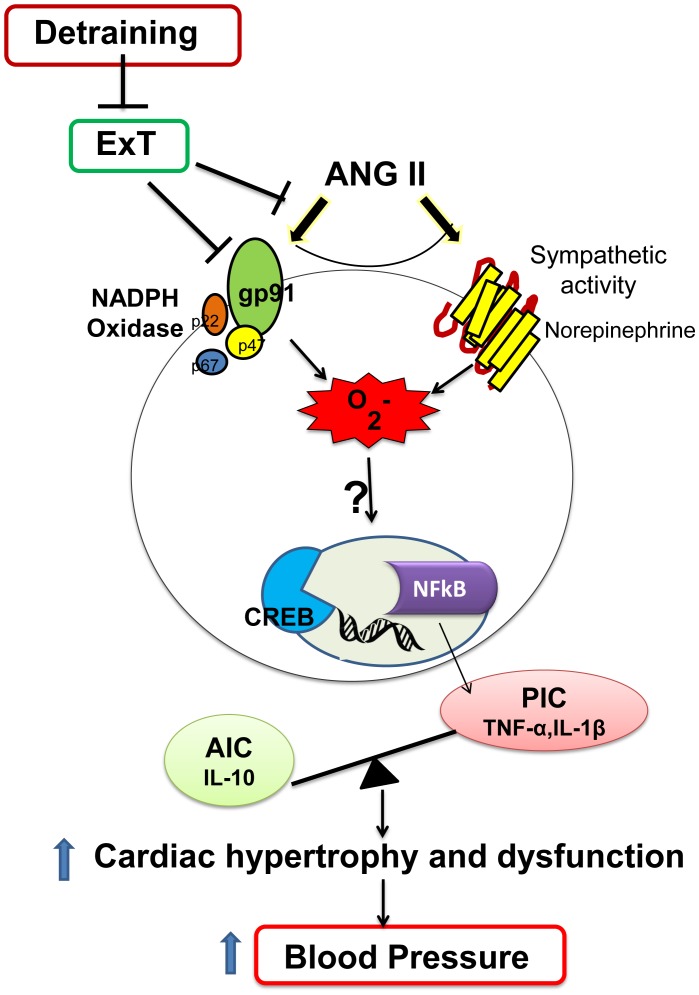
A schematic depicting the proposed pathways of effects of exercise training and detraining on AngII-induced hypertensive response. Lines with arrow represent ‘activation’ and lines with no arrow represent ‘inhibition’. It has become clear from the past several years of research that an increased production of PICs in response to overactivated RAS within the cardiovascular regulatory centers of the brain (such as paraventricular nucleus) causes increased sympathetic outflow leading to increased arterial pressure and cardiac remodeling in experimental models of hypertension. At the cellular level, PICs activate reactive oxygen species which in turn can activate various intracellular signaling pathways, including that of NFκB. Activation of NFκB induces gene transcription of PICs fostering a positive feedback mechanism, and eventually leading to the progression of hypertension. A growing body of evidence suggests that the beneficial effects of exercise in hypertension could be attributed to reduced PICs, improved cellular redox homeostasis, and downregulation of NFκB activity. A step further, in the present study, we demonstrated that transient cessation of exercise (2 weeks of detraining) abolishes the exercise-induced improvements in cardiac hypertrophy, cardiac function, anti-inflammatory cytokine (IL-10) and oxidative stress in the PVN of hypertensive rats, although, positive effects in MAP and PICs remains unchanged. Further studies are still warranted to unravel the effects of exercise and detraining on other components of the AngII-induced signaling pathway such as downstream transcription factors and sympathetic activity.

Given that exercise is recommended as a current guideline for the treatment of hypertension and non-compliance with the recommended treatment is a universal phenomenon, it is imperative to understand the cardiac and molecular changes associated with detraining. A few previous studies have examined the effects of detraining on heart and skeletal muscle of hypertensive and normal rats in relation to insulin sensitivity [1], [23]–[24].The results of the current study provides a greater insight in to how detraining can influence the mean arterial blood pressure, cardiac function, inflammatory cytokines, and redox status within the brain of hypertensive rats. Investigating the effects of exercise and detraining on other components of the AngII-induced signaling pathway such as downstream transcription factors and sympathetic activity could certainly be important perspectives of this study (Figure 7).

We have previously demonstrated that the beneficial effects of exercise in hypertension are mediated by reduced PICs, improved cellular redox homeostasis, and downregulation of NFκB activity (Figure 7). However, one can raise the possibility for role of RAS in exercise-induced beneficial effects as well. For instance, recent reports from our laboratory as well as others have showed that chronic exercise decreases circulating AngII and modulates vasoconstrictor and vasodilatory components of the RAS within the brain of spontaneously hypertensive rats [2] and heart failure rabbits [51]. Although we have not measured AngII levels within the PVN, the beneficial effects of exercise in AngII-induced hypertensive rats presently observed cannot be completely explained by a decrease in brain AngII because AngII was continuously infused in these rats through subcutaneously implanted minipumps. Nonetheless, it is becoming clear from all these studies that both AngII-dependent and –independent mechanisms of beneficial effects of exercise are taking place. However, further studies are still warranted to achieve deeper understanding of molecular mechanism involved in exercise-induced effects and how detraining modulates them. The understanding of the underlying molecular mechanisms and the time taken for each signaling pathway to lose adaptation induced by regular exercise will lead us to improve the current guidelines for the treatment of hypertension on the basis of scientific evidence.
